# Pyruvate Kinase Deficiency Causing Priapism

**DOI:** 10.1155/2023/6503311

**Published:** 2023-05-08

**Authors:** Vinay Hanyalu Shankar, Bharadwaj Adithya-Sateesh, Nicole Gousy, Girma Ayele, Freyr Petursson, Rediet Atalay, Miriam Michael

**Affiliations:** ^1^Department of Internal Medicine, Centinela Hospital Medical Center, Inglewood, CA, USA; ^2^American University of Antigua College of Medicine, Department of Medicine, Osbourn, Antigua and Barbuda; ^3^Department of Internal Medicine, Howard University, Washington, DC, USA; ^4^Department of Emergency Medicine, Centinela Hospital Medical Center, Inglewood, CA, USA; ^5^Department of Internal Medicine, University of Maryland Medical Center Midtown Campus, Baltimore, MD, USA

## Abstract

Pyruvate kinase deficiency (PKD) is an autosomal recessive defect of the enzyme pyruvate kinase (PK) which is involved in catalyzing a reaction that produces ATP in the glycolytic pathway. It is the most common defect of the glycolytic pathway associated with congenital anemia. Patients usually present with signs of chronic hemolytic anemia such as hyperbilirubinemia, splenomegaly, reticulocytosis, and gallstones; the presentation can vary by age. Diagnosis is usually made by demonstration of decreased PK enzymatic activity in a spectrophotometric assay and on the detection of mutations in the PK-LR gene. Management strategies vary from full splenectomies to hematopoietic stem cell transplants with gene therapies with transfusions and administration of PK-activators coming in between. Thromboembolic complications do occur in patients with splenectomy, but there are not much data regarding this for patients with PKD. We present a case of a patient with PKD who demonstrated priapism to be a thromboembolic complication. This differs greatly as priapism has been frequently reported in patients with other chronic hemoglobinopathies such as sickle cell disease, thalassemia, and G6PD with and without splenectomy. While it is still unclear how splenectomies can result in thrombotic events in PKD, there does appear to be a correlation between splenectomies with resultant thrombocytosis with increased platelet adhesion.

## 1. Introduction

Red blood cells lack mitochondria and a nucleus and use the glycolytic pathway for energy metabolism as RBCs. The reaction catalyzed by pyruvate kinase is the second ATP-generating step of the glycolytic pathway and is responsible for producing nearly 50% of the total ATP [1] Enzyme deficiencies can lead to decreased intracellular ATP levels, which possibly reduce RBC deformability. The ability to deform is an essential feature of red blood cells that enables them to travel through even the smallest capillaries of the human body.

Pyruvate kinase deficiency (PKD) is an autosomal recessive defect of the enzyme pyruvate kinase (PK). It is the most common defect of the glycolytic pathway associated with congenital anemia [2]. Two genes PK-M and PK-LR present on chromosomes 15 and 1q21, respectively, encode for four different PK isozymes [1, 2]. The PK isozymes M1 and M2 are encoded by PK-M of which M2 is the major isozyme, particularly of erythroid precursors, and the isozymes PK-L (liver) and PK-R (red blood cells (RBC)) are encoded by PK-LR [1, 2]. As erythroid precursors differentiate, the major isozyme switches from PK-M2 to PK-R [1, 2]. Hepatocytes have the capacity to synthesize proteins and maintain some residual PK-M2 activity and are unaffected in PKD [2]. The disease is most commonly due to missense mutations in PK-LR; therefore, mature RBCs expressing PK-R are affected more than their hepatocyte counterparts [1]. The disease has a prevalence of 3–8/1,000,000 but is not precisely defined given the rarity of the disease and broad spectrum of presentation [2]. PKD should be suspected in patients presenting with signs of chronic hemolytic anemia such as hyperbilirubinemia, splenomegaly, reticulocytosis, and gallstones; the presentation can vary by age [2]. Diagnosis is usually made by demonstration of decreased PK enzymatic activity by lysing RBCs in a spectrophotometric assay [2] and on the detection of mutations in the PK-LR gene. Management strategies vary from full splenectomies to hematopoetic stem cell transplants with gene therapies with transfusions and administration of PK-activators coming in between [2]. A few cases of thromboembolic events after splenectomy have been described in the literature in patients with PKD. We present a case of a patient with PKD who presented with priapism.

## 2. Case Presentation

A 21-year-old male with a past medical history of type 1 diabetes mellitus and pyruvate kinase deficiency presented to the emergency department with a chief complaint of a painful erection. The symptom first started at about 2:30 AM in the morning causing him to wake up from his sleep. He has had similar episodes which had resolved spontaneously within one to two hours; occasionally, he would take a warm shower or do jumping jacks which speeded the resolution. When waiting and repeating these manouveurs did not resolve the episode, he proceeded to the emergency room at 4:00 PM in the evening. On presentation, the patient had severe 9/10 nonradiating pain exacerbated by movements of any sorts. The patient was tachycardic and afebrile; the other vital signs were within normal limits. Physical exam showed a rigid penis that was tender to light palpation. Laboratory evaluation was significant for a WBC count of 18800/*μ*L with a neutrophilic predominance (78.9%), hemoglobin of 9.4 g/dL (the patients baseline was usually between 9 g/dL–10 g/dL) with an MCV of 112.6 fL. His platelet count was 491000/*μ*L. He was also found to be hyperglycemic with blood glucose of 274 mg/dL. Other significant lab findings included an obstructive pattern in his liver function tests with an aspartate aminotransferase (AST), alanine aminotransferase (ALT), and alkaline phosphatase of 75 IU/L, 81 IU/L, and 154 IU/L, respectively. The patient was given 4 mg morphine for pain and 1 L of normal saline. An 18-gauge needle was used and 35 cc of blood was aspirated from the corpus cavernosum on both sides following which 200 mg of phenylephrine was injected and a compression dressing was placed. On reevaluation, he required another aspiration followed by phenylephrine injection following which his symptoms resolved. The patient was discharged from the emergency department of note; the patient does not exactly recall when he was first diagnosed with pyruvate kinase deficiency but mentions it was during early childhood. He had a splenectomy in the second decade of his life and has been appropriately vaccinated. The patient was asked to follow up with a hematologist for his PKD.

## 3. Discussion

Pyruvate kinase deficiency (PKD) is an autosomal-recessive enzyme defect in the PK-LR gene on chromosome 1q21 [2]. This defect results in a significant reduction of ATP generated in red blood cells, especially considering the glycolytic pathway results in over 50% of the cell's total ATP [1]. Despite there being four isolated tissue-specific variants of PK isoenzymes, the clinical symptoms of PKD are solely restricted to that of the hematologic system [1]. Since RBCs heavily depend on glycolysis for ATP production, the absence of abnormal PK activity subsequently results in a loss of RBC membrane plasticity, cellular dehydration, and premature destruction of RBCs in the spleen and liver [2]. Additionally, one study by Kanno et al. reported an inverse correlation between functioning PK activity with quantities of apoptotic erythroid progenitors in the spleen [3]. This revealed that altered PK activity affects RBC maturation, possibly due to decreased ATP availability. Therefore, patients with PKD have inherently ineffective erythropoiesis. Lastly, decreased PK enzymatic activity leads to an increase in its upstream product, 2, 3-diphosphoglycerate (2,3-DPG), which, in elevated levels, induces a rightward shift in the hemoglobin-oxygen dissociation curve, leading to generally tolerable anemia [4, 5].

The presentation of PKD includes clinical signs of chronic hemolytic anemia, such as gallstones (especially black-pigmented), splenomegaly, jaundice, anemia, fatigue, shortness of breath, and bone pain [2, 6]. Laboratory results will typically show indirect hyperbilirubinemia, reticulocytosis, and hyperferritinemia [2]. Since the differential diagnosis of PKD also involves other congenital hemolytic disorders, the diagnosis of PKD is mainly that of exclusion, in addition to proven reduced PK enzymatic activity via analysis of RBC lysates by spectrophotometric assay [2, 5, 6].

Management of PKD relies on supportive care based on individualized patient symptoms [4]. Since symptoms are so widely ranging between patients, treatment regimens require personalization per patient that might differ during their lifespan. Frequent red blood cell transfusions with adjuvant chelation therapy are commonly required in those who are severely anemic, especially in young children with PKD [1, 2]. Splenectomy has been shown to increase the average hemoglobin levels by approximately 1–3 g/dl and can lessen or eliminate the need for transfusions [1]. Notably, thromboembolism has been reported as a complication following splenectomy, especially in those with hereditary chronic hemolytic anemia; however, there are scarce reports of thrombotic events associated with PKD [7]. In the previous case reports reporting a thrombotic event post-splenectomy, the event occurred years after the splenectomy, contrasting with the patient described above as only recently having a splenectomy and having numerous episodes of priapism.

While it is still unclear how splenectomies can result in thrombotic events in PKD, there does appear to be a correlation between splenectomies with resultant thrombocytosis with increased platelet adhesion. Disruptions to the RBC membrane can result in numerous changes to surface RBC thrombogenic phospholipids [7]. Compared to other inherited causes of chronic hemolytic anemia, however, PKD does not involve potentially thrombotic cellular structural changes that can lead to postsplenectomy thrombosis, such as Heinz bodies [7]. Therefore, for those with PDK postsplenectomy, thrombotic events are induced by chronic hemolysis alone. It is thought that chronic hemolysis of abnormal RBCs with reduced PK activity will result in the exposure of thrombophilic inner red cell membranes. The thrombotic inner red blood cell membranes, which would have been normally destroyed by the spleen, are then allowed to cause thrombotic events within the vasculature [7]. Therefore, this patient's multiple episodes of priapism might have been the result of chronic hemolysis of abnormal RBCs in addition to a recent splenectomy allowing for continued exposure of the thrombophilic inner RBC membranes.

In previous studies of thrombotic events in the setting of PKD, there have been very few reports of the development of recurrent priapism as a presentation for a thrombotic event. This differs greatly as priapism has been frequently reported in patients with other chronic hemoglobinopathies such as sickle cell disease, thalassemia, and G6PD with and without splenectomy [8]. However, there have also been reports of postsplenectomy priapism in those without chronic hematological disease, owing to resulting thrombocytosis secondary to the procedure [7]. This brings up the question of whether the thrombocytosis as a result of the splenectomy is the underlying reason for the recurring priapisms or is it the thrombophilia as a result of chronic hemolysis secondary to PKD to blame. Due to the rarity of PKD itself and reports of thrombotic events in PKD being even more scarce the true nature of this event remains unknown. Therefore, more research into PKD being a cause of recurring thrombotic events such as this is necessary.

## 4. Conclusion

Pyruvate kinase deficiency (PKD) is an autosomal recessive defect of the enzyme pyruvate kinase (PK) which is involved in catalyzing a reaction that produces ATP in the glycolytic pathway. Apart from glycolysis, PK has also been shown to play a role in RBC maturation. The disease usually manifests with symptoms of chronic hemolytic anemia, and diagnosis is made by measuring enzymatic activity. Management is usually based on the severity of the symptoms of each patient and is tailored to each patient on a case-by-case basis. Options include transfusions, gene therapy, and splenectomy. Splenectomies have been associated with the occurrence of thromboembolic complications, but the exact pathogenesis is rather unclear for patients with PKD. We presented a case of a patient who presented with priapism as a thromboembolic event postsplenectomy which varies greatly when compared to other chronic hemolytic states where patients usually present with priapism without splenectomy. The true nature of thromboembolic complications especially priapism is unknown in patients who have had a splenectomy, and further research into this is warranted.
